# Nucleoli cytomorphology in cutaneous melanoma cells – a new prognostic approach to an old concept

**DOI:** 10.1186/s13000-017-0675-7

**Published:** 2017-12-29

**Authors:** Piotr Donizy, Przemyslaw Biecek, Agnieszka Halon, Adam Maciejczyk, Rafal Matkowski

**Affiliations:** 1 Department of Pathomorphology and Oncological Cytology, Wroclaw Medical University, ul. Borowska 213, 50-556 Wroclaw, Poland; 2 Faculty of Mathematics and Information Science, Warsaw University of Technology, Koszykowa 75, 00-662 Warsaw, Poland; 3 Department of Oncology and Clinic of Radiation Oncology, Wroclaw Medical University, pl. Hirszfelda 12, 53-413 Wroclaw, Poland; 4 Lower Silesian Oncology Centre, pl. Hirszfelda 12, 53-413 Wroclaw, Poland; 5 Department of Oncology and Division of Surgical Oncology, Wroclaw Medical University, pl. Hirszfelda 12, 53-413 Wroclaw, Poland

**Keywords:** Nucleolus, Melanoma, Prognosis

## Abstract

**Background:**

The nucleolus is an organelle that is an ultrastructural element of the cell nucleus observed in H&E staining as a roundish body stained with eosin due to its high protein content. Changes in the nucleoli cytomorphology were one of the first histopathological characteristics of malignant tumors. The aim of this study was to assess the relationship between the cytomorphological characteristics of nucleoli and detailed clinicopathological parameters of melanoma patients. Moreover, we analyzed the correlation between cytomorphological parameters of nucleoli and immunoreactivity of selected proteins responsible for, among others, regulation of epithelial-mesenchymal transition (SPARC, N-cadherin), cell adhesion and motility (ALCAM, ADAM-10), mitotic divisions (PLK1), cellular survival (FOXP1) and the functioning of Golgi apparatus (GOLPH3, GP73).

**Methods:**

Three characteristics of nucleoli – presence, size and number – of cancer cells were assessed in H&E-stained slides of 96 formalin-fixed paraffin-embedded primary cutaneous melanoma tissue specimens. The results were correlated with classical clinicopathological features and patient survival. Immunohistochemical analysis of the above mentioned proteins was described in details in previous studies.

**Results:**

Higher prevalence and size of nucleoli were associated with thicker and mitogenic tumors. All three nucleolar characteristics were related to the presence of ulceration. Moreover, microsatellitosis was strongly correlated with the presence of macronucleoli and polynucleolization (presence of two or more nucleoli). Lack of immunologic response manifested as no TILs in primary tumor was associated with high prevalence of melanoma cells with distinct nucleoli. Interestingly, in nodular melanoma a higher percentage of melanoma cells with prominent nucleoli was observed. In Kaplan-Meier analysis, increased prevalence and amount, but not size of nucleoli, were connected with shorter cancer-specific and disease-free survival.

**Conclusion:**

(1) High representation of cancer cells with distinct nucleoli, greater size and number of nucleoli per cell are characteristics of aggressive phenotype of melanoma; (2) higher prevalence and size of nucleoli are potential measures of cell kinetics that are strictly correlated with high mitotic rate; and (3) high prevalence of cancer cells with distinct nucleoli and presence of melanocytes with multiple nucleoli are features associated with unfavorable prognosis in patients with cutaneous melanoma.

The preliminary results of this study was presented during the 29th European Congress of Pathology (Amsterdam, The Netherlands; 2–6 September 2017) and the abstract was published in the congress materials [1].

## Background

The nucleolus is an organelle that is an ultrastructural element of the cell nucleus observed in H&E staining as a roundish body stained with eosin due to its high protein content. In mammalian cells, nucleoli are present only in the interphase and are not observed during mitosis [2–4]. The nucleoli’s main function is the synthesis of ribosomal RNA (rRNA), that is why they are referred to as the ribosome factory. Additionally, the nucleolus is a specific sequestration/storehouse of proteins which under physiological conditions serve their role in the nucleoplasm. It was shown that the Cdc14 phosphatase is sequestered in yeast cell nucleoli and released to the cytoplasm during anaphase, being the key point of cell cycle progression [5]. Other biochemical functions of the nucleoli include their role in maintaining three-dimensional organization of chromatin in the nucleus [6]. It was demonstrated that perinucleolar chromatin is enriched in Snf2h, catalytic subunit of protein complex which is involved in chromatin remodeling that is necessary for normal replication of heterochromatin of exceptionally packed structure [7]. In addition to ribosome production, the nucleolus is also involved in the biogenesis of ribonucleoprotein particles independently from the synthesis of ribosome subunits – assembly of the signal recognition particle (SRP) [8–10], modification of U2 and U6 spliceosomal small RNA [11, 12] and assembly of specific mRNPs (small nuclear ribonucleoproteins) [13].

Based on the electron microscope analysis of the ultrastructure, the nucleolus has three major components: fibrillar center (FC), dense fibrillar component (DFC) and granular component (GC) [3]. Ribosomal genes actively engaged in transcription are located dominantly in two components: FC and DFC, thus being a functionally active unit of the nucleolus that is involved in rRNA synthesis. Granular component is responsible for maturation of ribosomal subunits [14, 15].

Changes in the nucleoli cytomorphology (their entry to the cells and the evaluation of their size) were one of the first histopathological characteristics of malignant tumors, along with abnormal mitotic figures, thickened and irregular nuclear membrane and coarse chromatin [4]. Eosinophilic macronucleoli are characteristic e.g. for melanoma, serous adenocarcinoma, epithelioid sarcoma, prostatic adenocarcinoma or Hodgkin lymphoma. It must be stressed, however, that only the presence of nucleoli (micronucleoli, less often macronucleoli) is a feature of metabolically active cells. Therefore, the presence of nucleoli in the cells does not allow us to qualify the analyzed cells as malignant cancer cells – e.g. normal macrophages and hepatocytes may present with clear nucleoli which only means they are functionally active as regards protein synthesis, and not that they are undergoing cancer transformation. It must also be underlined that the cells of some clinically extremely aggressive cancers do not have prominent nucleoli (or even do not have them at all), e.g. desmoplastic small round cell tumor (DSRCT) and small cell neuroendocrine carcinoma – in our opinion it may be related with extremely high Ki67 proliferative index and high mitotic rate in the case of these two cancers, which suggests that a high percentage of cancer cell population is in the active phase of mitotic division, which excludes the presence of nucleoli within these cells.

The aim of this study was to assess the relationship between the cytomorphological characteristics of nucleoli and detailed clinicopathological parameters of melanoma patients with survival analysis. Moreover, we analyzed the correlation between cytomorphological parameters of the nucleoli and immunoreactivity of the selected proteins related, among others, with the regulation of EMT (SPARC, N-cadherin), cell adhesion and motility (ALCAM, ADAM-10), mitotic divisions (PLK1), cellular survival (FOXP1) and the functioning of Golgi apparatus (GOLPH3, GP73).

## Methods

### Patients

Our study group was composed of 96 cutaneous melanoma patients treated at the Lower Silesian Oncology Center in Wroclaw, Poland, diagnosed in 2005–2010. Patients were enrolled in the study based on the availability of their medical documentation and tissue material, which included paraffin blocks and histopathology slides. Comprehensive clinical data was retrieved from the archival medical records, and data concerning the diagnostic and therapeutic procedures used was sourced from the cancer outpatient clinic at the Lower Silesian Oncology Center and Lower Silesian Cancer Registry, as well as Civil Register Office. The study was reviewed and approved by the ethical committee of the Wroclaw Medical University, Wroclaw, Poland.

Records were reviewed for clinical and pathological data (age and gender, primary tumor location, tumor stratification according to AJCC (pT), presence or absence of nodal (pN) and distant (pM) metastases, information on disease recurrence and SLNB procedures (Table 1).

**Table 1:** Correlations between characteristics of nucleoli in malignant melanocytes and clinical parameters

Clinical parameters	Characteristics of nucleoli								
	Size			Presence			Number		
	Low	High	*p* value	Low	High	*p* value	Low	High	*p* value
Age in years (21–79)^a^ mean, 57 ± 15.4; median, 58			0.463			0.883			0.480
Gender^b^									
Female	46	11	0.618	25	32	0.532	42	15	0.464
Male	29	10		20	19		32	7	
Primary tumor location^c^									
Head/neck	10	4	0.927	5	9	0.295	11	3	0.722
Extremities	33	8		20	21		30	11	
Hand/ft	2	1		0	3		3	0	
Trunk	30	8		20	18		30	8	
Primary tumor (pT)^a^									
pT1	28	6	**0.022**	21	13	**0.025**	27	7	0.233
pT2	17	0		10	7		15	2	
pT3	15	9		6	18		15	9	
pT4	15	6		8	13		17	4	
Regional lymph nodes status (pN)^b^									
No metastases (pN-)	64	17	0.735	38	43	1.000	64	17	0.326
Metastases present (pN+)	11	4		7	8		10	5	
Distant metastases (pM)^b^									
No metastases (pM-)	71	20	1.000	44	48	0.363	72	19	0.076
Metastases present (pM+)	4	1		1	4		2	3	
Sentinel lymph node biopsy status (SNLB)^b^ (55 patients)									
No metastases (SNLB-)	37	8	1.000	23	22	1.000	37	8	1.000
Metastases present (SNLB+)	8	2		5	5		8	2	
Recurrence^b^									
No	64	18	1.000	39	43	0.778	63	19	1.000
Yes	11	3		6	8		11	3	

^a^ *p* value of Wilcoxon two sample test

^b^ *p* value of Fisher’s exact test

^c^ *p* value of chi^2^ test;

Statistically significant results (*P* < 0.05) are in bold text

### Histopathological parameters

Archival formalin-fixed and paraffin-embedded tumor specimens were analyzed. Specifically, all hematoxylin and eosin-stained sections of the primary tumor were examined independently by two pathologists who reported data such as Breslow thickness, Clark level, histologic type, mitotic rate (number of mitotic figures per 1 mm^2^), presence of ulceration, lymphangioinvasion, microsatellitosis, intensity of tumor infiltrating lymphocytes (TILs) and microscopic evidence of regression (Table 2).

**Table 2:** Correlations between characteristics of nucleoli and presence of intranuclear vacuoles in malignant melanocytes and histopathological parameters

Histopathological parameters	Characteristics of nucleoli								
	Size			Presence			Number		
	Low	High	*p* value	Low	High	*p* value	Low	High	*p* value
Breslow thickness^a^									
≤ 1 mm	28	6	**0.022**	21	13	**0.025**	27	7	0.233
1.01–2.00 mm	17	0		10	7		15	2	
2.01–4.00 mm	15	9		6	18		15	9	
> 4 mm	15	6		8	13		17	4	
Clark level^a^									
II and III	55	8	**0.003**	35	28	**0.032**	48	15	0.806
IV and V	20	13		10	23		26	7	
Histologic type^b^			0.316			**0.012**			0.511
Superficial spreading melanoma (SSM)	53	11		36	28		50	14	
Nodular malignant melanoma (NMM)	20	9		9	20		21	8	
Acral-lentiginous melanoma (ALM)	2	1		0	3		3	0	
Mitotic rate^a^									
0	40	4	**0.006**	28	16	**0.004**	37	7	0.152
≥ 1	35	17		17	35		37	15	
Ulceration^c^									
No	46	6	**0.012**	31	21	**0.008**	45	7	**0.026**
Yes	29	15		14	30		29	15	
TILs									
No	14	3		4	13		13	4	0.585
Non-brisk	24	7	0.887	13	18	**0.038**	22	9	
Brisk	37	11		28	20		39	9	
Microsatellitosis^c^									
No	74	17	**0.008**	44	47	0.363	73	18	**0.010**
Yes	1	4		1	4		1	4	
Lymphatic invasion									
No	55	16	1.000	36	35	0.248	58	13	0.098
Yes	20	5		9	16		16	9	
Tumor regression^c^									
No	71	18	0.340	42	47	1.000	69	20	1.000
Yes	4	3		3	4		5	2	

^a^ *p* value of Wilcoxon two sample test

^b^ *p* value of chi^2^ test

^c^ *p* value of Fisher’s exact test;

Statistically significant results (*P* < 0.05) are in bold text

### Evaluation of nucleoli

**Fig. 1:**
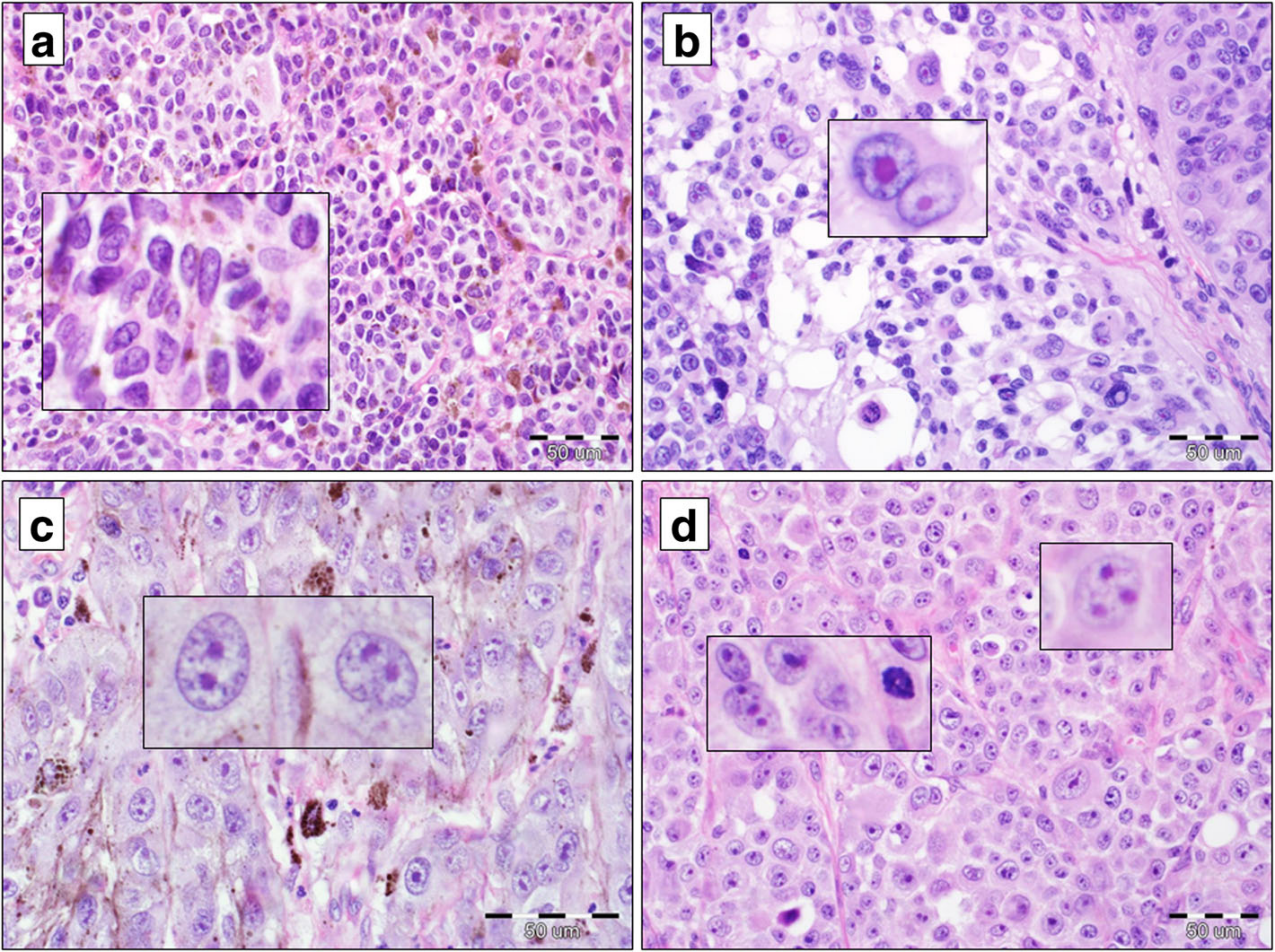
Cytomorphology of nucleoli in cutaneous melanoma cells. Lack of nucleoli in neoplastic cells ((**a**), H&E staining, × 400; insert: H&E staining, × 600). Small number of melanoma cells with visible nucleoli ((**b**), H&E staining, × 400; insert: in higher magnification two cells with macro- and micronucleoli, H&E staining, × 600). High representation of cancer cells with distinct nucleoli ((**c**), H&E staining, × 600; insert: prominent binucleolization (two nucleoli per one melanoma cell), H&E staining, × 600). High percentage of melanoma cells with prominent nucleoli ((**d**), H&E staining, × 400; insert: polynucleolization of melanoma cells (three nucleoli per one melanoma cell, H&E staining, × 600)

**Fig. 2:**
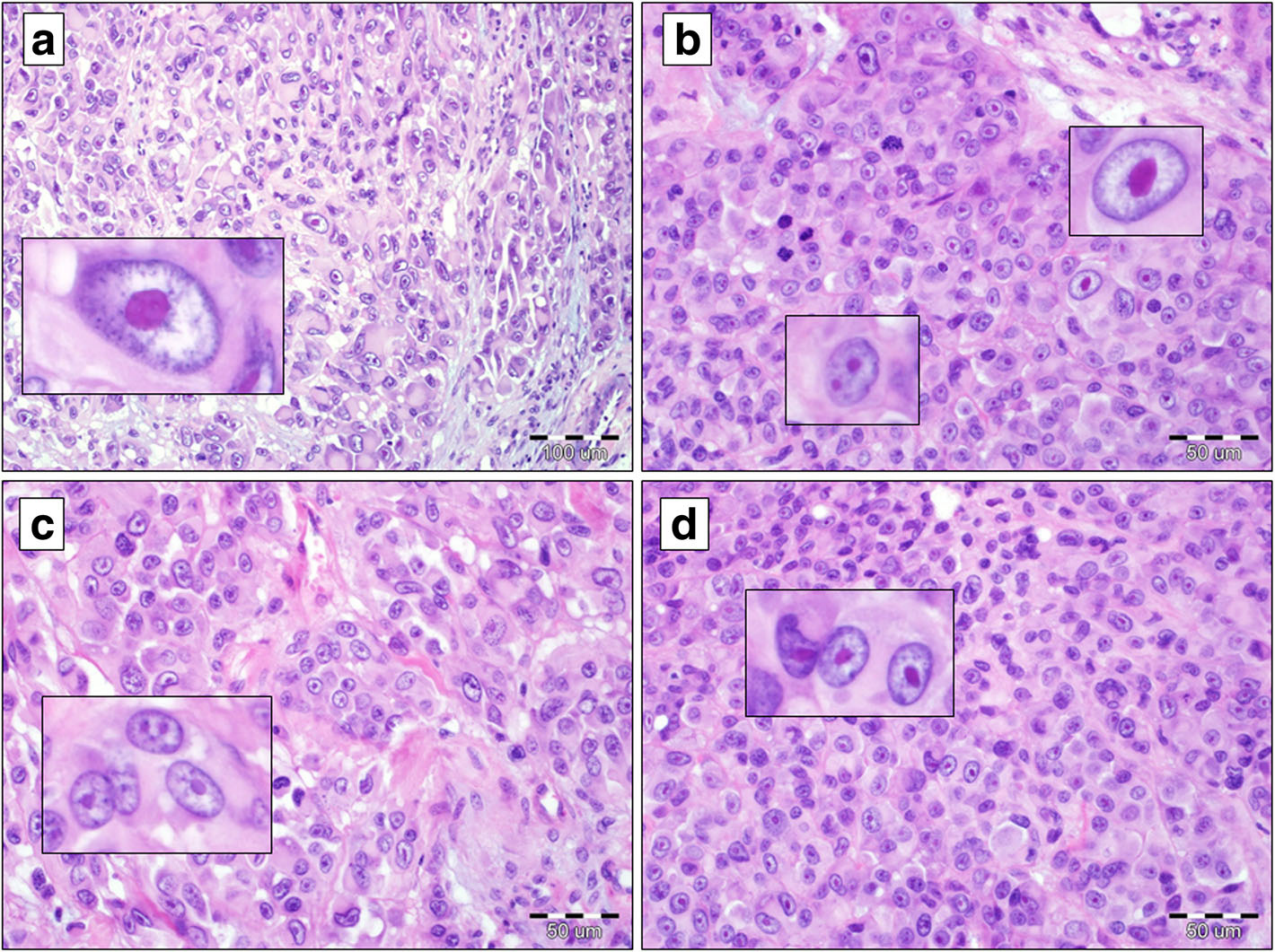
Cytomorphology of nucleoli in cutaneous melanoma cells. Prominent macronucleoli in melanoma cells ((**a**), H&E staining, ×400; insert: eosinophilic roundish macronucleolus, H&E staining, ×600). Melanoma cells of high heterogeneity in the context of cytomorphological parameters of the nucleoli – cells with micronucleoli and cells with prominent macronucleoli present ((**b**), H&E staining, ×400; insert: H&E staining, ×600). High representation of cancer cells with distinct nucleoli with the feature of binucleolization ((**c**), H&E staining, ×400; insert: H&E staining, ×600; (**d**) staining, ×400; insert: eosinophilic micronucleoli, H&E staining, ×600);

Three cytomorphological parameters were introduced to characterize the nucleoli. The presence of nucleoli refers to the global/total evaluation of the presence of nucleoli in the melanoma cell nuclei using the following grouping algorithm: 0: no nucleoli in melanoma cells, 1: small number of cells with the presence of nucleoli (≤ 20% of melanoma cells in the analyzed single H&E stained specimen of the primary tumor), 2: high percentage of cells shows the presence of nucleoli (> 20% of melanoma cells in the analyzed single H&E stained specimen of primary tumor). Nucleolus size refers to the size of the analyzed nucleoli (0: no nucleoli in melanoma cell nuclei, 1: micronucleoli present (inconspicuous nucleoli), 2: macronucleoli present (prominent nucleoli). Nucleoli number refers to the number of nucleoli in melanoma cell nuclei (0: no nucleoli in melanoma cell nuclei, 1: single micro- or macronucleolus in the nucleus, 2: two or more nucleoli per one nucleus of melanoma cell) Figs. 1 and 2.

### Immunohistochemistry

Immunohistochemistry on formalin-fixed paraffin embedded tissue was done as described previously [16–20].

### Statistical analysis

Statistical analysis was performed using the R language (available online: https://www.r-project.org/). Continuous variables like the age or proportions of lymphocytes were summarized with the use of the mean, median, min and max values. As regards the analysis of correlation of individual nucleolar parameters, the following dichotomous divisions of the study group were introduced for the purposes of statistical analysis: (1) presence of nucleoli: no nucleoli in melanoma cells or small number of cells with the presence of nucleoli (≤ 20% of melanoma cells in the analyzed single H&E stained specimen of the primary tumor) versus high percentage of cells shows the presence of nucleoli (> 20% of melanoma cells in the analyzed single H&E stained specimen of primary tumor); (2) nucleolus size: no nucleoli in melanoma cell nuclei or micronucleoli present (inconspicuous nucleoli) versus macronucleoli present (prominent nucleoli); (3) nucleoli number: no nucleoli in melanoma cell nuclei or single micro- or macronucleolus in the nucleus versus two or more nucleoli per one nucleus of melanoma cell.

For cancer-specific overall survival (CSOS) and disease-free survival (DFS), we performed log-tests and Kaplan-Meier curves; all such analyses were conducted with the survival package for R. To assess the relation between dichotomized cytomorphological parameters of nucleoli in melanoma cells and continuous variables, the Wilcoxon two-sample test was used. The relation of cytomorphological parameters of nucleoli with binary variables was assessed by exact Fisher’s exact test while the relation with other categorical variables was assessed by chi-square test. All relations were summarized by a suitable *p*-value, and all *p*-values smaller than 0.05 were considered as significant.

## Results

### Correlations with clinical parameters

A statistically significant correlation was shown between high percentage of melanoma cells with the presence of observable and/or clear nucleoli and higher advancement of primary tumor (pT) (*p* = 0.025). Additionally, also the size of nucleoli themselves – the presence of macronucleoli was correlated with a more advanced cancer process (*p* = 0.022). No other significant correlations were observed between cytomorphological parameters of the nucleoli and other clinical characteristics e.g. the status of regional lymph nodes or the presence of distant metastases (Table 1).

### Correlations with histopathological parameters

Higher prevalence and size of nucleoli were associated with thicker primary tumor in the context of Breslow and Clark scales (p = 0.025 and p = 0.022, respectively). Moreover, these cytomorphological parameters of nucleoli were observed in primary tumor with high mitotic rate (*p* = 0.004 and *p* = 0.006, respectively). A statistically significant correlation was shown between high percentage of melanoma cells with the presence of observable nucleoli and nodular melanoma (*p* = 0.012). All three nucleolar characteristics were related to the presence of ulceration (*p* = 0.008, p = 0.012 and *p* = 0.026, respectively). Moreover, microsatellitosis was strongly correlated with the presence of macronucleoli and polynucleolization (presence of two or more nucleoli) (p = 0.008 and *p* = 0.010, respectively). Interestingly, lack of immunologic response manifested as no TILs in primary tumor was associated with high prevalence of melanoma cells with distinct nucleoli (*p* = 0.038) (Table 2).

### Impact of nucleoli cytomorphology on long-term survival – Kaplan-Meier analysis

**Fig. 3:**
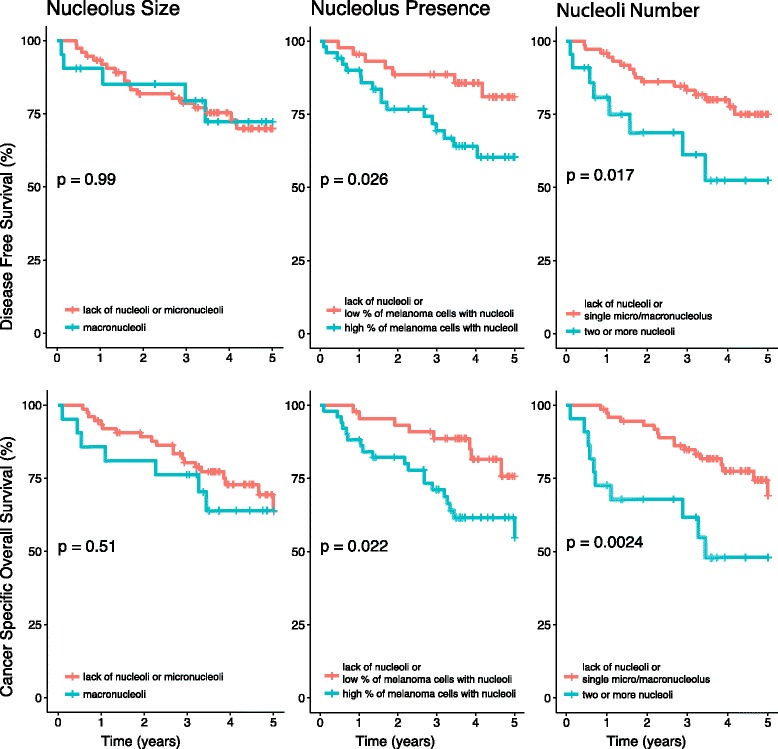
Kaplan-Meier analysis of the prognostic impact of nucleoli parameters in cutaneous melanoma patients. Increased prevalence and number, but not size of nucleoli, were connected with significantly shorter disease-free and cancer-specific overall survival

In Kaplan-Meier analysis, increased prevalence and number, but not size of nucleoli, were connected with significantly shorter disease-free and cancer-specific overall survival (Fig. 3).

### Correlations between cytomorphology of nucleoli and expression parameters of selected proteins

A statistically significant correlation was demonstrated between the presence of macronucleoli and increased number of nucleoli (polynucleolization) and decreased expression of GOLPH3 protein in tumor-associated macrophages (*p* = 0.034 and *p* = 0.042, respectively). GOLPH3 immunoreactivity in melanoma cells did not show statistically significant correlations with the presence of macronucleoli and polynucleolization. No significant correlations were shown between cytomorphological parameters of the nucleoli and expression of proteins related with the regulation of EMT (SPARC, N-cadherin), cell adhesion and motility (ALCAM, ADAM-10), regulation of mitotic divisions (PLK1) or cellular survival (FOXP1) (data not shown).

## Discussion

In this study we revealed that higher prevalence and nucleolar hypertrophy were associated with thicker and mitogenic tumors. Presence of macronucleoli and polynucleolization (presence of two or more nucleoli) was strongly correlated with microsatellitosis which is postulated as an unfavorable prognostic factor in melanoma patients. All three nucleolar characteristics were related to the presence of ulceration – one of the most important histopathological bad prognostic parameter. Interestingly, lack of immunologic response manifested as no TILs in primary tumor was associated with high prevalence of melanoma cells with distinct nucleoli. Kaplan-Meier analysis confirmed that increased prevalence and number, but not size of nucleoli, were connected with shorter cancer-specific and disease-free survival.

The nucleolus that is actively engaged in transcription, and is morphologically manifested as eosinophilic, hypertrophic, prominent nucleolus (sometimes also a few nucleoli in a single cell) is strictly related with a high translational potential of a cell and is an indicator of the cell’s high demand for proteins (e.g. proto-oncogene proteins). Prominent nucleoli are specific measures of cellular kinetics, as they are a morphological equivalent of the cell preparing for mitotic division which always requires a massive number of regulatory, structural and functional proteins. We may also state that prominent nucleoli observed in a routine H&E staining are specific mirrors of extremely numerous, abnormally intensified cytobiochemical changes that occur in cancer cells. In our study we showed a statistically significant correlation between high mitotic rate and a higher prevalence and size of nucleoli, which confirms that the changes of nucleoli morphology are strictly correlated with increased proliferation potential. In other words, morphological changes of the nucleoli are basically the result of increased demand for ribosome synthesis which is a feature of cells with high proliferation potential. In our opinion, the evaluation of cytomorphology of the nuclei could be a more informative parameter than mitotic rate, since cytomorphological changes of the nucleoli occur at a much earlier stage of cancer transformation – the presence of mitotic figures in cancer melanocytes is the final stage and clue of cancer development and the nucleoli help us identify an increased proliferation potential at a much earlier stage. We should highlight the fact that the assessment of the nucleolar features is of academic interest but does not currently warrant a change in routine histopathological practice, as it does not seem to independently predict prognosis in melanoma in addition to the already well-established prognostic parameters. It could be very promising cytomorphological parameter assess only with cooperation with well-established parameters, such as: Breslow thickness, mitotic rate and ulceration. Interestingly, studies conducted by Lee et al. [21] concerning hepatocellular carcinoma showed that nucleolar hypertrophy appears to be independent of cell proliferation – most of hepatocytes in dysplastic tumors with enlarged nucleoli did not show increased cell proliferation. It may have been related with early phase of accumulation of molecular disorders in dysplastic foci that precede the development of invasive carcinoma.

In our study we observed a statistically significant correlation between the presence of macronucleoli and increased number of nucleoli (polynucleolization) and decreased expression of GOLPH3 protein in tumor-associated macrophages (TAMs). Having no factual insight into the role of GOLPH3 in TAMs, we only speculate that the reduced GOLPH3 immunoreactivity in TAMs may be associated with stable prooncogenic M2 phenotype [19]. Due to the lack of clinical and molecular data careful functional investigations are needed to explore the roles of tumor-associated macrophages in melanoma.

Studies conducted over recent several years concerning molecular biology of the nucleoli have revealed a few molecular mechanisms which might explain the processes of nucleolar hypertrophy and their increased number in cancer cells. One of them is concerned with *c-Myc* gene proto-oncogene whose translation product is necessary for cell-cycle entry [22]. Its direct effect on ribosome biogenesis was showed which involves direct enhancement of RNA polymerase and transcription activity. The main mechanism involves binding to specific consensus elements of rDNA and recruiting the selectivity factor 1 (SL1) to the rDNA promoter [3, 23, 24]. SL1 is the key factor that in cooperation with UBF (upstream binding factor) enables rDNA transcription by recruiting RNA polymerase I [25]. Additionally, c-Myc oncoprotein regulates transcription of many proteins directly involved in ribosome biogenesis such as cyclin D and E [26].

The second mechanism involves the effect of mutations in *TP53* gene which result in inactivation and accumulation of p53 protein in the nucleus. Wild-type (non-mutated) p53 binds directly to selectivity factor SL1 thus preventing the formation of SL1-UBF complex which is necessary for RNA polymerase I recruitment to the rRNA gene promoter, and finally inhibits RNA Pol I transcription [3, 27]. Mutated inactive p53 loses its function of a negative controller of rRNA transcription, significantly increasing ribosome biogenesis.

An important molecular mechanism behind a considerable increase in nucleoli volume and number in cancer cells is related with inactivation of pRB protein. Active, nonphosphorylated pRB, through binding with UBF inhibits rRNA synthesis [28, 29]. During the progression of cell cycle, phosphorylation of pRB by cyclin-dependent kinases 2 and 4 results in freeing UBF and E2Fs, which directly induces increase in rRNA transcription, morphologically manifesting as nucleolar hypertrophy and polynucleolization [30].

Prognostic importance of the nucleoli morphology was widely studied over the recent years [4]. It must be stressed, however, that most authors analyzed the presence of AgNORs and not the cytomorphology of the nucleoli assessed based on H&E staining. In line with our observations, in the vast majority of cancers the larger the size of the nucleolus (when examining AgNORs areas), the worse the prognosis of the disease [31]. A similar correlation was observed in melanoma [32]. PubMed literature research did not bring any paper that would evaluate the nucleoli morphology and its prognostic significance in melanoma based on a routine H&E staining. Our studies have showed that the evaluation of cytomorphology of the nucleoli does not need to involve special histochemical techniques – we are able to obtain reliable prognostic information from a routine H&E staining.

## Conclusions

To conclude, (1) high representation of cancer cells with distinct nucleoli, greater size and number of nucleoli per cell are characteristics of aggressive phenotype of melanoma; and (2) higher prevalence and size of nucleoli are potential measures of cell kinetics that are strictly correlated with high mitotic rate.

## Abbreviations

ADAM10: A disintegrin and metalloproteinase domain-containing protein 10
AgNORs: Argyrophylic nucleolar organizer regions
AJCC: American Joint Committee on Cancer
ALCAM: Activated leukocyte cell adhesion molecule
CSOS: Cancer-specific overall survival
DFC: Dense fibrillar component
DFS: Disease-free survival
DSRCT: Desmoplastic small round cell tumor
FC: Fibrillar center
FOXP1: Forkhead box P1
GC: Granular component
GOLPH3: Golgi phosphoprotein 3
GP73: Golgi protein 73
H&E: Hematosylin and eosin
PLK1: Polo-like kinase 1
SL1: Selectivity factor 1
SNLB: Sentinel lymph node biopsy
SPARC: Secreted protein acidic and cysteine rich
SRP: Signal recognition particle
TILs: Tumor-infiltrating lymphocytes
UBF: Upstream binding factor
